# Nurses Entitled to Registration

**Published:** 1921-08-13

**Authors:** 


### THE MATRONS' AND SISTERS' DEPARTMENT.

### NURSES ENTITLED TO REGISTRATION.

The long-delayed Rules framed by the General Nursing Council for England and Wales are now in our hands. The old question of what constitutes in the eyes of the law a trained nurse has been answered, and it remains to take stock of what has been achieved.

In the first place, it is hardly possible to avoid a little pæan of thanksgiving that State registration has been delayed in this country until such time as it could inflict injustice on none to limit the title of trained nurse to women who have taken a course of institutional training. If we remember rightly, the standard of qualification at the time when the Royal British Nurses' Association issued its regulations provided for the admission of all, irrespective of training, who could produce evidence of sound morals and of *bonâ-fide* practice as a nurse for a set period. The immense gulf which lies between nurses who have received formal training in the nursing of sick persons, even though in too small an institution and for too short a time, and those who have scrambled into a hazy knowledge of nursing procedure, under the guidance of the doctor in charge of the case, is very perceptible. The latter can now no longer lay claim to be nurses at all. None who have not been trained under trained nurses are to be registered. That is the crux of the whole matter.

The highest class of existing nurses to be placed on the Register are those who hold certificates of not less than three years' training in a general hospital having one or more resident medical officers, or in a Poor-law infirmary recognised by the Minister of Health for the training of superintendent nurses, and in both cases recognised by the Council for training. It will be observed that there is no mention of any particular number of beds as conferring a title to be a recognised training-school. It is the presence of a resident medical officer which is the criterion, and this distinction will be generally recognised as just. The training capacity of a hospital cannot be judged entirely by its size. It is the class of cases received, not merely their number, which tests the value of the ward work which the nurse has to perform. And many a comparatively small institution becomes under keen professional stimulus a centre of modern methods and progress infinitely better adapted to train nurses than a large institution which has become a backwater.

Nurses who have had three years' training in general hospitals or infirmaries unprovided with a resident medical officer will also be admitted to the general part of the Register during the two years of grace, but they must satisfy the Council that they have adequate knowledge and experience of the nursing of the sick. We conceive this requirement to mean testimony forwarded from their matron as to the kind of work in which they have been exercised. It does not imply examination of the applicant, but rather inquiry into the advantages she has enjoyed.

There is a comparatively small number of nurses who have received but one year's training in hospitals and infirmaries "approved by the Council." It used to be the custom in some large institutions to offer some such shortened form of training, usually by payment, and to refuse the name of trained nurse to women who took their year's training in all good faith, and have since made good by *bonâ-fide* practice as nurses, would be manifestly unfair. These nurses are to be admitted to the general part of the Register. Their training, short though it be, has been usually carried out under the best conditions, and some have earned a high reputation.

Next we have the class of nurses with mixed training who can produce certificates of two years' training in general hospitals for children, hospitals for infectious diseases, or hospitals for women, together with evidence of one subsequent year's training in a general hospital or Poor-Law infirmary approved by the Council. Certain hospitals and infirmaries entered into agreements with each other to supplement special training facilities by grafting on to it a period of general training, and the result was to produce efficient nurses with a good grasp of their work. It is generally believed that this system will be further developed when the General Nursing Council issues its Regulations respecting the training of future nurses. The nurses who have been trained under these conditions have a strong claim to State registration, as have also the superintendent or head nurses appointed by the Ministry of Health prior to November 1, 1919.

It must be evident to all that the bogey which has so long disturbed the peace of trained nurses has been effectually exorcised by the publication of the General Council's Rules. The General Register will not contain the names of any who have not received careful preparation for performing their nursing duties. Untrained matrons and sisters are now non-existent. Each nurse who satisfies any of the above requirements will have passed through the hands of a trained superintendent and will have received the sign manual of training even though she may lack many of the advantages enjoyed by her more modern compeers.

